# Drug Carrier for Photodynamic Cancer Therapy

**DOI:** 10.3390/ijms160922094

**Published:** 2015-09-14

**Authors:** Tilahun Ayane Debele, Sydney Peng, Hsieh-Chih Tsai

**Affiliations:** 1 Graduate Institute of Applied Science and Technology, National Taiwan University of Science and Technology, 106 Taipei, Taiwan; E-Mail: tilish4ayane@yahoo.com; 2 Department of Chemical Engineering, National Tsing Hua University, 300 Hsinchu, Taiwan; E-Mail: sunchips4u@hotmail.com

**Keywords:** photodynamic therapy, photosensitizers, cancer cells, nanoparticles, biodegradable, organic nanocarrier, inorganic nanocarrier

## Abstract

Photodynamic therapy (PDT) is a non-invasive combinatorial therapeutic modality using light, photosensitizer (PS), and oxygen used for the treatment of cancer and other diseases. When PSs in cells are exposed to specific wavelengths of light, they are transformed from the singlet ground state (S_0_) to an excited singlet state (S_1_–S_n_), followed by intersystem crossing to an excited triplet state (T_1_). The energy transferred from T_1_ to biological substrates and molecular oxygen, via type I and II reactions, generates reactive oxygen species, (^1^O_2_, H_2_O_2_, O_2_*, HO*), which causes cellular damage that leads to tumor cell death through necrosis or apoptosis. The solubility, selectivity, and targeting of photosensitizers are important factors that must be considered in PDT. Nano-formulating PSs with organic and inorganic nanoparticles poses as potential strategy to satisfy the requirements of an ideal PDT system. In this review, we summarize several organic and inorganic PS carriers that have been studied to enhance the efficacy of photodynamic therapy against cancer.

## 1. Introduction

### 1.1. Principle and Mechanism of Photodynamic Therapy

**Figure 1:**
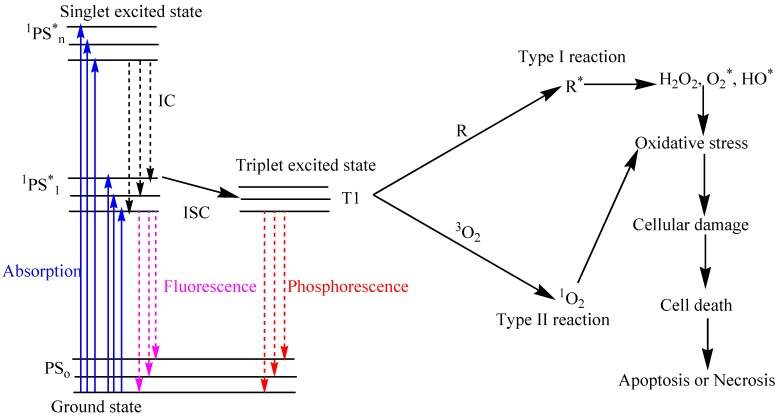
Modified Jablonski diagram depicting the process of photodynamic therapy. When PSs in cells are exposed to specific wavelengths of light, they are transformed from the singlet ground state (S_0_) to an excited singlet state (S_1_–S_n_), which is followed by intersystem crossing to an excited triplet state (T_1_). Abbreviation: IC: internal conversion; ISC: intersystem crossing; PS: photosensitizer; ^1^PS*: Singlet excited photosensitizer; T1: Triplet excited state; R: biological substrate; R*: oxidized biological substrate; ^1^O_2_: Singlet oxygen; H_2_O_2_: hydrogen peroxide; O_2_*: superoxide; HO*: hydroxyl radical.

**Figure 2:**
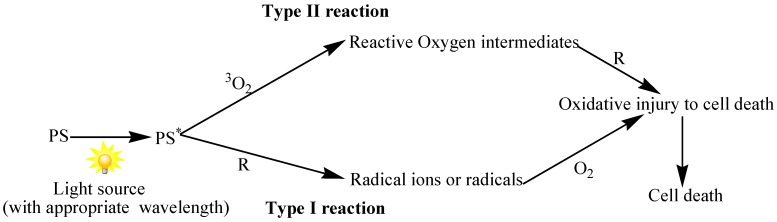
Pathways of Type I and Type II reaction of light absorbing photosensitizer. After light activation of the photosensitizer (PS) at ground state, activated form of PS (PS*) can follow two separate pathways. Type I reaction occurs when the excited molecule reacts with organic substrates (R) and produce radical ions or radicals. Type II reaction takes place when the energy is transferred from the excited photosensitizer to molecular Oxygen (^3^O_2_) to form reactive oxygen intermediates. These intermediates react rapidly with their surroundings including cell wall, cell membrane, peptides, and nucleic acids.

Photodynamic therapy (PDT) is a non-invasive therapeutic method that is effective against cancers, bacteria, virus, and other microbes [1,2]. The three key players of PDT include light, photosensitizer (PS), and oxygen. After light exposure at a specific wavelength, the PS is excited from the ground state (S_0_) to the excited singlet state (S_1_) and undergoes intersystem crossing to a longer-lived excited triplet state (T_1_), as shown in Figure 1 [3,4]. Photosensitizers may undergo one of two types of reactions at the T1 state [5], including type I reaction where excited molecules react with the substrate to generate active radicals that in turn react with biological components such as plasma membrane, peptides, proteins, and nucleic acids [6,7], or Type II reaction, which involves the transfer of energy from the activated photosensitizers to oxygen molecules in the excited single state [8] (Figure 2). Both reactions occur instantaneously and the likelihood of their occurrence is dependent on the type of photosensitizer used, the abundance of substrate and oxygen, as well as the binding affinity of the sensitizer for the substrate [9]. On the other hand, PDT is restricted to local and proximal cells due to short half-life of reactive oxygen species (ROS) [9]. In order for the system to be effective, damages incurred from PDT must exceed that repairable by the cell repair mechanism [10].

Energy transferred from T_1_ to biological substrates (R) and molecular oxygen, via type I and II reactions, generates ROS (^1^O_2_, H_2_O_2_, O_2_*, HO*). This causes cellular damage that can lead to tumor cell death through either necrosis or apoptosis.

### 1.2. Anti-Tumor Activity of PDT

**Figure 3:**
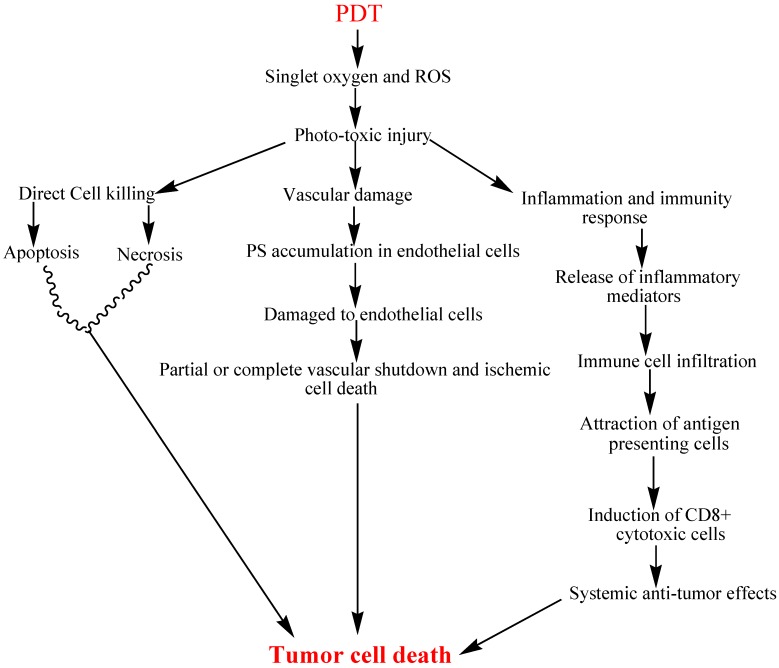
Schematic view of the anti-tumor effects of PDT.

Mechanisms through which PDT may exhibit anti-tumor activity have been explored in various studies and include (a) direct tumor cell damage; (b) damage to the vasculature; and (c) inducing inflammatory and immune response which briefly summarized in Figure 3 [11,12,13,14].

#### 1.2.1. Direct Tumor Cell Kill

Generally, the site of photodamage is dependent on preferential accumulation of PSs in tumor cells, where subsequent irradiation activates a cascade of photochemical reactions that lead to the generation of singlet oxygen (^1^O_2_) and other highly reactive oxygen species [15]. These species are accountable for the oxidation of biomolecules such as proteins, peptides, amino acids, and lipids. Depending on the location of the PSs, irreversible damages are done to the cellular plasma membrane and vital subcellular membranes such as lysosome, mitochondria, endoplasmic reticulum (ER), Golgi apparatus, and nuclear membrane [16,17].

Different signaling pathways may be triggered during PDT to mediate either apoptotic or necrotic cell death. The type of cell death observed is dependent on the type of cell, PS concentration, and the identity and dose of light [13,18]. Previous study has shown that mitochondria and ER localization of PSs normally lead to the apoptotic pathway [19]. On the other hand, the necrotic pathway may be initiated when PSs are localized in the plasma membrane or lysosomes [20]. If photosensitizing compounds accumulate in the cell membrane, cells will die through the necrotic pathway due to loss of cell membrane integrity [16,21]. In addition, stimulation of PSs in the lysosomes interrupts the lysosomal membrane, thereby releasing proteases that activate Bid, a pro-apoptotic member of the Bcl-2 family, and triggering the mitochondrial apoptotic pathway [22]. Some researchers believe that PDT-related lethality is mainly attributed to mitochondrial membrane damage, which disrupts membrane potential and releases cytochrome C into the cytoplasm. Cytochrome c binds to Apf-1 and procaspse-9 to elicit the formation of a complex protein called apoptosome, which triggers various caspases (caspase-9, -2, -3, -6 and -7) and hydrolytic enzymes, resulting in the cleavage of cellular proteins, fragmentation of DNA, and eventually cell death [23]. Furthermore, numerous studies have shown that p53 is recruited to the endoplasmic reticulum (ER) and ER-mitochondrial contact sites during cell stress and enhances ER Ca^2+^ levels through ER-Ca^2+^-ATPase activity, which results in mitochondrial permeability transition due to Ca^2+^ overload in the mitochondrial matrix [24,25]. This causes mitochondrial swelling and rupture of the outer mitochondrial membrane, releasing pro-apoptotic factors, such as cytochrome C, and activating different caspases that lead to apoptosis [24]. Giorgi, C. *et al.* demonstrated Ca^2+^-dependent p53-mediated cancer cell apoptosis induced by phototherapy [26]. The team combined the dorsal skinfold chamber technique with intravital microscopy to elucidate the involvement of p53 in controlling intracellular Ca^2+^ signals and apoptosis in three-dimensional tumor masses *in vivo*.

#### 1.2.2. Vascular Damage

Angiogenesis, the synthesis of new blood vessels, is vital to cancer cell proliferation and metastatic spread because newly formed vessels provide ample supply of nutrients and oxygen [27]. Hence, selectively damaging microvasculature and inhibiting angiogenesis will effectively increase the treatment efficacy of PDT [28]. Destruction of blood vessels during and after PDT is noticeable through vessel constriction, vessel permeability, and leukocyte adhesion [29]. Fluid and macromolecular leakage occurs in vessels shortly after light treatment and produces tissue edema [30]. These events contribute to blood flow stasis and produce regional tissue hypoxia [31]. The destruction of tumor cells is believed to be a result of this hypoxia and the accompanying deprivation of nutrients. Chen *et al.* examined the effect of vascular-targeting PDT on vascular barrier function both in subcutaneous (S.C.) and orthotopic MatLyLu rat prostate tumor models and endothelial cells *in vitro*, using photosensitizer verteporfin [32]. The group found that vascular-targeting photodynamic therapy permeabilizes blood vessels through the formation of endothelial intercellular gaps, which were likely induced by endothelial cell microtubule depolymerization after vascular photosensitization. The loss of endothelial barrier function can ultimately result in tumor vascular shutdown.

#### 1.2.3. Inflammatory and Immune Response

PDT’s effect on the immune system may be described as either immunosuppressive or immunostimulatory [33]. Inflammatory reactions arising from PDT is reflected as a vital event in the development of anti-tumor immune response [34]. The principle characteristic of the inflammatory process is the release of inflammatory mediators at the treated region. PDT-treated cells showed enhanced production of danger signals such as heat shock proteins (HSP), complement proteins, Eicosanoids, chemokines and cytokinase such as TNF-α, IL-6, IL-1, and different transcription factors including NF-κB and AP-1, which upsurges antigen presentation by dendritic cells (DCs) and recruitment of antigen-specific cytotoxic T lymphocytes (CTLs) [35,36,37,38].

### 1.3. Photosensitizer

Photosensitizers (PS) are natural or synthetic chromophore-containing compounds that absorb light at a specific wavelength and can induce chemical or physical changes of another chemical entity to generate reactive oxygen species (ROS) [39]. An ideal PDT photosensitizer possesses the following characteristics [40,41]: (1) It is readily available in pure form with known chemical composition and low dark toxicity; (2) The PS is stable and soluble in aqueous media; (3) It may be rapidly excreted from the body to reduce systemic toxicity at the end of treatment; (4) It exhibits high quantum yield in cell inactivation, which is frequently mediated by singlet oxygen (^1^O_2_); (5) High molar absorption coefficient in the 600–1000 nm range because endogenous molecules such as hemoglobin and melanin have strong absorption below 600 nm, thereby limiting the effective penetration depth of light in target tissues. Furthermore, water absorbs many photons above 1000 nm and reduces availability towards chromophores and longer wavelengths are energetically inadequate for the production of ^1^O_2_; (6) There should be maximum quantum yield of triplet formation and the triplet state should extensively exist in order to rapidly react with neighboring target molecules. Currently, only a few PSs have been approved for clinical use while others are under clinical trial [41,42,43,44]. PSs currently on the market or under clinical trials are summarized in Table 1 and Table 2, respectively.

**Table 1:** Clinically approved PDT photosensitizers in oncology.

Photosensitizer	Approved Application
Porfimer sodium (Photofrin)	Used in the treatment of early and late-stage lung cancers, esophageal cancer, bladder cancer, early stage cervical cancer, and malignant and nonmalignant skin diseases. It is also being considered as potential therapy against Kaposi’s sarcoma, Barrett’s esophagus with high-grade dysplasia, psoriasis, and cancers of the head, brain, neck and breast [45,46]
5-Aminolevulinic acid or ALA (Levulan)	US FDA approved for non-oncological PDT treatment of actinic keratosis in 1999. Its potential PDT applications extend to Bowen’s disease, basal cell carcinoma, and other diseases. ALA can also be used to detect tumors in bladder, skin, lung, and gastrointestinal tract [47,48,49,50]
Methyl aminolevulinate (Metvixia)	Approved by the US FDA in 2004 for treatment of actinic keratosis [51,52]
*Meta* tetra(hydroxyphenyl) chlorin (Foscan)	Treatment of neck and scalp cancer with *m*-THPC was approved in Europe, and the drug was used successfully for the treatment of breast, prostate, and pancreatic cancers [53,54,55]
*N*-aspartyl chlorin e6 (NPe6, Laserphyrin)	Approved for the treatment of fibrosarcoma, liver cancer, brain cancer, and oral cancer. Approved in Japan in 2003 to treat lung cancer [46]
Benzoporphyrin derivative monoacid ring A (Visudyne)	In 1999, US FDA approved Visudyne for age-related macular degeneration in ophthalmology [41]
*n*-Hexyl ester of ALA (Cysview)	Approved in 2010 by the US FDA for the diagnosis of bladder cancer [56]

words in Bold in the brackets indicate trademark of the photosensitizers.

**Table 2:** PDT photosensitizers under clinical Trials.

Photosensitizers	Potential indication
Hypocrellin A	White lesions of vulva and keloid cases, antiviral activity against human immunodeficiency virus type 1 and age-related macular degeneration [57,58]
Pheophorbide-a	Early stage lung cancer, superficial head and neck cancer and human uterine cancer [59,60]
Chlorin e6	Superficial squamous cell carcinoma of the lung, human nasopharyngeal and bladder carcinomas [61,62,63]
Methylene blue	Basal cell carcinoma, Kaposi’s sarcoma and Melanoma [64,65]
Hypericin	Bladder cancer, nasopharyngeal carcinoma cells [66,67]
Phthalocyanine	Cutaneous/subcutaneous lesions from diverse solid tumor origins [68]
Rose Bengal	Metastatic melanoma [69]
HPPH: 2-(1-Hexyl-oxyethyl)-2-devinyl pyropheophorbide-alpha	Equine periocular squamous cell carcinoma, rodent colon carcinoma and xenografts of human glioma [70,71]

### 1.4. Light Source in PDT

Light source is another important parameter in PDT and include laser (argon laser and argon-pumped dye laser, metal vapor-pumped dye laser, solid state laser, diode laser, *etc.*) and non-laser sources/lamps (quartz halogen lamps, metal halide lamps, xenon arc lamps, phosphor-coated sodium lamp, fluorescent lamps, *etc.*) as comprehensively reviewed by Brancaleon and Moseley [72]. The efficacy of PDT depends on factors such as wavelength, tissue penetration, dose, pulsing, position, state of the cells/tissues, and absorption properties of the photosensitizers [73,74]. The principle requirement is ample light illumination at the appropriate wavelength which can sensitive PSs and penetrate deep into the target/tumor tissues [75]. Since PDT is most effective toward tumors that that can be reached directly by light or light delivery devices such as optic fibers [76], highest PDT efficacy is attained when light delivery is homogenous and adequate throughout the target tumor tissue volume.

## 2. Photosensitizer Delivery

Tumor blood vessels exhibit unique features not observed in normal vessels including extensive angiogenesis, extensive extravasation (vascular permeability), defective vascular architecture, and impaired lymphatic clearance from the interstitial space of tumor tissues [77]. Tumor vascular permeability and impaired lymphatic clearance allows nanoparticles to accumulate at the tumor site, a phenomenon known as enhanced permeability and retention (EPR) effect. Traditional small molecule drugs and PSs, on the other hand, lack selectivity and are known to distribute throughout normal tissues [78]. Nano-sized carriers have additional advantages over conventional low molecular weight agents such high loading capacity, protection from degradation, long circulating time, selective targeting, and controlled release [79]. Solubility and effective targeting are the two crucial factors in designing photosensitizer for PDT [80,81]. To increase the overall efficacy of PDT, solubility and selectivity may be enhanced through the use of nanocarriers or surface modification using specific ligands such as monoclonal antibodies, peptides, or polyethylene glycols [82,83].

### 2.1. Nanoparticles-Based PSs Delivery in PDT

Nanoparticles, defined as having one dimension that measures 1000 nanometer or less, have distinct physical, chemical, magnetic, and structural characteristics that allow them to broadly used in drug delivery, medical imaging, sensing, diagnosis, and therapy [83,84]. Nanoparticle-based PS delivery systems satisfy most of the requirements for an ideal PS, as described in the previous section [85]. In these systems, PSs are either encapsulated in or immobilized to nanoparticles through covalent/non-covalent interactions [86]. A major advantage of nanosizing is the yield of high surface to volume ratio and the possibility of high drug loading [87]. More specifically, nanotechnology is attractive in PDT for three major reasons: (1) Targeting potential enhances PS concentration at the desired site and reduces toxic effects toward normal tissues/cells; (2) Nanoparticles can improve the solubility of hydrophobic PSs; and (3) Constant rate of PS delivery results in zero-order release kinetics, thereby maintaining a constant therapeutic dose at the site of action [86,87,88]. These nanosized PS carriers can be further classified into organic and inorganic nanoparticles.

## 3. Organic Nanoparticles

Organic nanoparticles can be defined as solid particles composed of organic compounds such as lipids, proteins, polysaccharides, or polymers and bear the advantage of having low toxicity and versatility of carrying a diverse selection of drugs [89,90].

### 3.1. Liposomes

**Figure 4:**
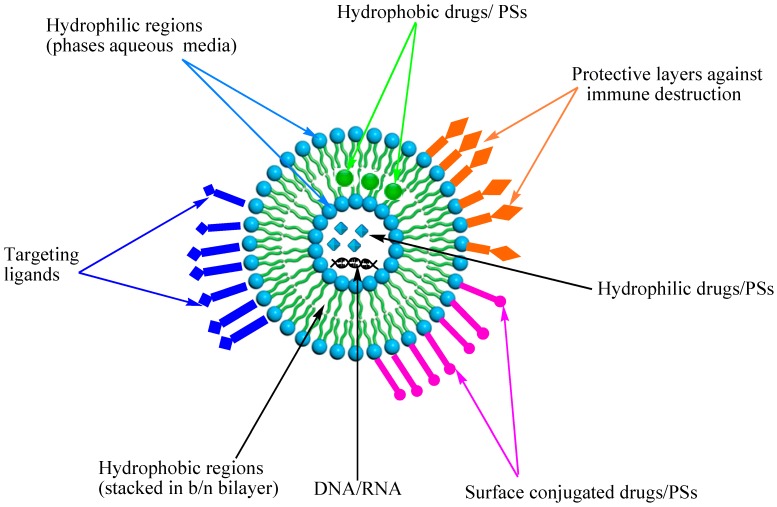
Modified schematic representation of liposome, with drug entrapped in the aqueous phase and within the bilayer.

Liposomes are synthetic lipid vesicles made up of one or more concentric phospholipid bilayers as depicted in Figure 4 [84]. Phosphatidyl inositol, phosphatidyl serine, and phosphatidyl choline are the most commonly used lipids to prepare liposomes and may be used alone or in combination with other substances to vary the liposomes’ physio-chemical and biological properties such as size, drug loading capacity, and permeability [91]. Liposomes have both a hydrophilic and hydrophobic region, thereby permitting the encapsulation and transport of both hydrophilic and hydrophobic PS [92]. Several studies have shown strong evidence that liposomal formulations of PS is effective in PDT. Jiang *et al.* reported that PDT using liposome-encapsulated Photofrin resulted in more significant destruction of U87 glioma brain tumor as compared to Photofrin loaded in dextrose vehicles [93]. Selectivity towards tumor tissues was demonstrated with liposomal Photofrin while concentration of the PS was similar in normal tissues after treatment with liposomal and free Photofrin. Similar results have been observed in different tumor cell models using different photosensitizers [94,95].

Due to rapid disintegration and short plasma half-life, conventional liposomes are less effective in establishing elevated tumor-to-normal tissue ratios [96]. Surface modifications have been explored to produce so-called long-circulating liposomes with enhanced plasma stability [97]. PSs have noticeable photoactivity in their monomeric form, yet because most PSs are water insoluble and strongly aggregate in aqueous media, their photosensitizing efficiency is significantly diminished. Liposomal formulation effectively prevents the aggregation of PSs and in turn enhances their photoactivity [98]. Wang *et al.* reported that the liposomal hypocrellin A, a hydrophobic PS, exhibited lesser degree of aggregation and higher tumor-selective accumulation in comparison to a suspension of the same PS in dimethyl sulfoxide/saline [99].

While conventional liposomes are swiftly removed from circulation after systemic administration by macrophages of the reticulo-endothelial system (RES) [100], the circulatory half-life of liposomes may be significantly extended by surface modification [101]. The most commonly used ligand is poly(ethylene glycol) (PEG), a hydrophilic polymer that curtails recognition and uptake by the RES to maintain effective blood concentration. These modified liposomes exhibit protein resistance, minimum toxicity and non-immunogenicity [102,103]. Nawalany *et al.* reported the use of pegylated liposomes to reduce tetrakis (4-hydroxyphenyl) porphyrin (p-THPP) aggregation in the treatment of prostate adenocarcinoma cell line (DU 145) and human colon adenocarcinoma cell line (HCT 116) [103]. Furthermore, selective accumulation of p-THPP in cancer cells and increased efficiency of PDT were observed. Later in 2012, Nawalany *et al.* evaluated the effect of PEG chain length on the photodynamic activity of a series of PEG-functionalized tetraarylporphyrin liposomes [104]. Results showed that that attachment of PEG chains ranging from 350 to 5000 Da resulted in pronounced reduction in the dark cytotoxicity of the parent porphyrin against DU 125 and HCT 116. Cell viability tests further demonstrated that the phototoxicity of pegylated systems was highest with PEG chain of 2000 Da, which induced greater degree of apoptosis in both cell lines when compared to native or pegylated porphyrin delivered in solution. In general, several pharmaceutical companies are currently examining novel PS-delivering liposomes for the treatment of tumors [105]. Visudyne^®^, a liposomal formulation of Verteporfin, was approved in 2000 for the treatment of age-related macular degeneration and 2001 for the treatment of pathological myopia [106].

### 3.2. Polymeric Nanoparticles

Polymeric nanoparticles have been extensively studied for their chemical and physical properties and used widely to encapsulate a variety of drugs [107]. These polymeric nanoparticles are prepared from a variety of biocompatible polymers and can be formulated to transport PSs in a controlled and targeted fashion through further surface modification with specific ligands [108].

Polymeric nanocarriers may be prepared from both natural polymers, such as albumin [109], hyaluronic acid [110], and chitosan, [111], and synthetic polymers, such as poly acrylamide (PAA), poly lactic acid (PLA), poly glycolic acid (PGA), poly(lactide-*co*-glycolide) (PLGA), dendrimers, and hyperbranched polymers [112].

#### 3.2.1. Natural Polymeric Nanoparticles

Natural biodegradable polymers are attractive components in formulating PS transport nanoparticles due to their low cost, abundance, water solubility, biodegradability, biocompatibility, and potential for a multitude of chemical modifications [111,113].

##### Albumin

Albumin is the most abundant plasma protein and has been used widely for the preparation of nanocapsules and nanospheres due to its availability, biodegradability, nontoxicity, non-immunogenicity, hydrophilicity, and ease of preparation [114]. Furthermore, surface reactive groups such as amines, thiols, and carboxylic acids provide potential for functionalization. Human serum albumin (HSA) is a potential candidate for site-directed drug delivery due to simplicity in ligand attachment to the surface of the protein [115]. One of the major advantages of albumin nanoparticle is its high binding capacity for various dugs due to multiple drug binding sites [116]. Drugs can be loaded by electrostatic adsorption onto the surface of pre-formed nanoparticles, incorporated into the nanoparticle matrix, or covalently linked to the protein [117]. Drugs entrapped or conjugated to albumin nanoparticles can be released through hydrolysis, diffusion, or enzymatic degradation of nanoparticles [111]. Wacker *et al.* prepared 5,10,15,20-tetrakis(m-hydroxyphenyl) porphyrine (mTHPP) and chlorin (mTHPC) loaded HAS nanoparticles using the desolvation method [118]. The nanoparticle formulation protected the drugs from aggregation and oxidation while in solution form. After incubation with Jurkat cells, PS-loaded HAS efficiently generated singlet oxygen, which indicates that the PSs were released from nanoparticles upon cell uptake.

Chen *et al.* compared the phototoxicity of pheophorbide-HSA (Pheo-HSA) nanoparticles prepared by non-covalent drug adsorption at a molar loading ratio of 4:1 and crosslinked at 40% (PHSA40) and 100% (PHSA100) [119]. After 1 h of incubation with Jurkat cells (clone E6-1 human acute T-cell leukaemia), both formulations yielded minimum amount of singlet oxygen from intramolecular interactions. After 24 h of incubation and illumination, 71%, 85%, and 53% phototoxicity was observed for PHSA100, PHSA40, and free Pheo, respectively. Of the cell death accounted, 90% occurred via the apoptotic pathway. In 2010, Chen *et al.* performed a similar evaluation using mTHPC [120]. The loading ratio and degree of crosslinking were varied and photophysical properties of mTHPC-HSA such as fluorescence, fluorescence lifetime, and absorption were found to be primarily dependent on these two factors. Samples with low loading ratio (<17.8 µg/mg) exhibited strong interaction between mTHPC and HAS while samples with high loading ratio (>17.8 µg/mg) were predominantly stabilized by mTHPC-mTHPC interactions. In addition, the degree of cross-linking was found to influence photosensitizer-oxygen interaction and the oxygen concentration within the microenvironment is strongly affected by the degree of cross-linking. In highly crosslinked HAS particle singlet oxygen may be quenched, resulting in shortened singlet oxygen lifetime. Furthermore, high degree of crosslinking may prevent the adequate release of mTHPC upon cell uptake. Nevertheless, *in vitro* studies indicated that all samples generated singlet oxygen inside Jurkat cells.

##### Chitosan

Chitosan was first discovered in 1811 by Henri Braconnot and is the second most abundant biopolymer after cellulose [121]. It is cationic partially deacetylatd chitin (poly-*N*-acetyl glucosamine) derivative consisting of β-1,4-linked glucosamine (2-amino-2-deoxy-β-d-glucose) and minor amounts of *N*-acetyl glucosamine [122]. Chitosan is insoluble in water and organic solvents, but can be dissolved in mildly acidic solutions [123]. Chitosan is used for pharmaceutical applications due to its reactive functional groups, biocompatibility, gel forming capability, non-toxicity, high charge density, biodegradability, and mucoadhesiveness [124]. Cationic nature of chitosan allows the formulation of ionic complexes through electrostatic interactions between chitosan and anionic drugs [125]. Drug release from chitosan nanoparticles can be controlled through degree of deacetylation, which in turn affects polymer degradation by cell lysozymes [126]. Generally, decreasing degree of deacetylation corresponds to higher initial degradation.

Derivatives of chitosan typically exhibit faster degradation in comparison to free chitosan. In one study, increasing the molar ratio of glycolic acid to chitosan in poly(glycolic acid)-grafted chitosan increased the rate of degradation [127]. A number of other studies focused on the enhanced targeting ability and bioavailability of chitosan nanoparticles after modification [128]. Nam *et al.* synthesized, by partial derivatization of the free amino groups of glycol chitosan (GC) with 5β-cholanic acid, hydrophobically modified glycol chitosan (HGC) nanoparticles [129]. These HGC nanoparticles possessed tunable physiochemical properties, biocompatibility, low toxicity, tumor specificity, and fast cellular uptake through various routes, making them promising carriers for hydrophobic PSs. *In vitro* tests revealed that the accumulation of HGC nanoparticles in HeLa cells was significantly higher than that of native GC particles. Lee *et al.* prepared PS-loaded Chitosan nanoparticles (CNP) by self-assembly of amphiphilic glycol chitosan-5β-cholanic acid conjugates in an aqueous environment and subsequently loading protopor-phyrin IX (PpIX), to achieve high drug-loading efficiency (>90%) using a dialysis method [130]. *In vitro* studies showed that PpIX-CNPs were rapidly internalized by Squamous cell carcinoma (SCC7) with no dark cytotoxicity. CNPs exhibited high tumor specificity and therapeutic efficacy due to EPR effect and the production of cytotoxic singlet oxygen after irradiation, which resulted in irreversible cell death. One day post injection, PpIX-CNPs treated SCC7 tumor bearing mice exhibited 34% and 44% tumor volumes reductions compared to saline and PpIX-treated mice, respectively, after 1 h of irradiation. *In vitro* and *in vivo* results confirmed both tumor specificity and therapeutic efficacy of PpIX loaded nanoparticles in tumor-bearing mice. Drug loading method is another important parameter that should be considered because *in vitro* and *in vivo* characteristics may differ greatly depending on the method used. Lee *et al.* compared loaded (HGC-Ce6) and conjugated (GC-Ce6) chlorin e6-glycol chitosan nanoparticles prepared from self-assembled amphiphilic glycol chitosan-5-β-cholanic acid [131]. Ce6 was loaded physically by a simple dialysis method while chemical conjugation was performed to attach Ce6 to glycol chitosan. Both HGC-Ce6 and GC-Ce6 formed stable spheres in an aqueous environment and exhibited similar *in vitro* singlet oxygen generation efficacy under buffered condition. However, greater phototoxicity was observed with HGC-Ce6 due to rapid release of Ce6s in HGC-Ce6 (58% released within 5 h). On the other hand, *in vivo* evaluation using HT-29 colon adenocarcinoma tumor-bearing mice revealed that HGC-Ce6 did not efficiently accumulate in tumor tissues due to rapid release of the loaded drug, while GC-Ce6 exhibited prolonged circulation and efficient accumulation at target tumor tissues. The total photon count of GC-Ce6 in tumor tissues was approximately 6–7-fold higher than that of free Ce6 and HGC-Ce6. The final HT-29 tumor volume in mice treated with PDT using GC-Ce6 was approximately 60 mm^3^ 20 days post injection, which was significantly smaller than the tumor volumes after treatment using free Ce6 (760 mm^3^) and HGC-Ce6-treated mice (560 mm^3^).

Self-quenchable nanoparticles permit PSs to be distributed in their inactive form during systemic circulation to circumvent toxicities such as blood cell hemolysis and long-lasting cutaneous photosensitivity [132]. Once the nanoparticles are internalized into cancer tissues, PSs are released into the intracellular environment and recover their photosensitivity through a series of degradation processes. However, in order to attain maximum cytotoxicity, release kinetics and activation efficiency need to be considered [132]. Oh *et al.* designed a cancer-specific PS nanocarrier PheoA-*ss*-CNPs by conjugating pheophorbide a (PheoA) with glycol chitosan (GC) through reducible disulfide bonds [133]. In solution, PheoA-*ss*-CNPs can self-assemble into stable nanostructures with quenched photoactivity. After they are taken up by cancer cells, nanoparticles immediately dissociate by reductive cleavage of the disulfide linkers and undergo a dequenching process. Neither free CNPs nor free PheoA exhibited cytotoxicity in the absence of irradiation. However, after 20 min of irradiation, PheoA-*ss*-CNPs and free PheoA treatment resulted in 45% increase in cytotoxicity when compared to non-irradiated samples. Furthermore, conjugation through stable amide linkages instead of reducible disulfide bonds prevented the restoration of photoactivity in response to intracellular reductive conditions. PheoA-*ss*-CNP also exhibited prolonged blood circulation when compared to free PheoA, allowing tumor targeting by the EPR effect. Enhanced tumor accumulation increased the efficacy of PheoA-*ss*-CNP against HT29 tumor-bearing and shows that PheoA-*ss*-CNP is an attractive system for cancer PDT.

##### Hyaluronic Acid

Hyaluronic acid (HA) is a non-sulfated glycosaminoglycan (GAGs) that occurs naturally in high concentrations in various soft connective tissues such as synovial fluid, vitreous humor, skin, and the umbilical cord [134]. HA is a hetero polysaccharide with repeating units of d-glucuronic (d-Glu) acid and *N*-acetyl-d-glucosamine (d-GlcNAc) disaccharide [(1→3)-β-d-GlcNAc-(1→4)-β-d-Glu-] that was first isolated in 1934 from bovine vitreous humor by Meyer and Palmer [135,136]. For more than three decades, HA and its derivatives have been clinically used as medical products [137,138]. Major therapeutic applications of HA include ophthalmology [139,140], surgery and wound healing [141,142] and Orthopedic surgery and rheumatology [143].

HA-based nanoparticles are rapidly captured by RES and their receptors are highly expressed in the spleen, liver, and lymph nodes [144]. Currently, surface modification of HA with PEG is the most promising approach in reducing RES uptake. When used in a drug delivery system, HA can play the role of carrier and targeting agent at the same time, offering the opportunity for selectivity and release of cargo at the site of action [134,145]. However, the strong hydrophilic nature of HA renders it inappropriate for hydrophobic drug conjugation and as a result, the preparation methods in polar organic solvents have been developed to solve this problem [146]. Yoon *et al.* reported the use of tumor-targeting hyaluronic acid nanoparticles (HANPs) to carry hydrophobic Ce6 for simultaneous photodynamic imaging and therapy [145]. In the study, aminated 5-β-cholanic acid, PEG, and black hole quencher3 (BHQ3) were chemically conjugated to HA polymers and Ce6 was loaded by dialysis. The loading efficiency of Ce6-hyaluronic acid nanoparticles (Ce6-HANPs) was greater than 80%. The resulting nanoparticles were stable in aqueous conditions and were rapidly taken up by tumor cells. The nanoparticles were internalized by cells due to CD44 receptor binding and 4.1-fold higher fluorescence was observed in treated cells compared to untreated NIH3T3 30 min after incubation. Upon internalization, Ce6-HANPs were rapidly degraded by hyaluronidase, an abundant enzyme in the cytosol of tumor cells, enabling intracellular release of Ce6 at the tumor tissue. After intravenous injection, Ce6-HANPs efficiently accumulated at the tumor tissue via passive targeting and entered tumor cells through receptor-mediated endocytosis. With irradiation Ce6 released from the nanoparticles generated fluorescence and singlet oxygen inside tumor cells to effectively suppress tumor growth. Similarly, Li *et al.* prepared self-organizing HA nanogels through simple dispersion of acetylated HA-pheophorbide A (PheoA) conjugates (Ac-HA-PheoA) in aqueous solution [147]. The nanogels were auto quenched in PBS and their fluorescent intensity strongly correlated with the amount of PheoA with decreasing critical self-quenching concentration (CQC) associated with increased PheoA. After incubation cells, the degradation of HA by intracellular enzymes in compartments such as endosome and lysosome led to dequenching of the nanogel. Confocal imaging and FACS analysis showed that Ac-HA-PheoA nanogels were rapidly internalized into HeLa cells via a HA-induced endocytosis mechanism. These results indicate that HA based nanogels can potentially be applied to photodynamic therapy (PDT).

#### 3.2.2. Synthetic Polymer

Synthetic polymers provide the opportunity to develop new drug delivery systems with specific physiochemical and biological properties for specific applications through simple variation of building blocks or preparation methods [148]. Unlike natural polymers, synthetic polymer may be prepared with high reproducibility. A number of synthetic polymers are biodegradable due to the presence of ester, carbonate, amide, orthoester, urea, urethane, or anhydride linkages in their backbone [149]. Furthermore, biodegradability may be engineered into synthetic polymers by the introduction of such moieties [123].

##### PS loading of Polymeric Micelle

Although polymeric nanoparticles bear advantages and high solubilization potential, achieving maximum drug load still remains a challenge [150]. The most commonly used PS loading method into polymeric micelles include (i) oil-in-water (O/W) emulsion techniques [151]; (ii) water-in-oil-in-water (W/O/W) emulsion techniques [152,153,154]; (iii) direct dialysis [155]; (iv) co-solvent evaporation [156,157]; and (v) freeze-drying/lyophilization [158]. Among the above methods, O/W, direct dialysis, and co-solvent evaporation are well suited for the encapsulation of hydrophobic drugs, whereas W/O/W is often preferred for the encapsulation of more hydrophilic compounds [159].

##### Mechanisms of PS Release from Polymers

External physical and chemical signals such as temperature [160], pH [161], and ions [162] can alter polymer and carrier properties including volume, molecular interactions, solubility, conformation, and crystallinity [163], all of which in turn affect the drug release mechanism [164,165,166,167,168]. In the case of pH-sensitive polymers, their swelling is controlled by various factors such as charge and pKa of the ionizable monomers, hydrophilicity, crosslinking density, ionic strength, pH, and composition of the surrounding solution [169,170,171,172]. In general, PSs are released mainly from polymer nanoparticles through one of the following physico-chemical mechanisms [173,174].

(i) Enzymatic reaction that results in cleavage or degradation of the polymer at the site of delivery, thereby releasing PSs from the core.
(ii) Swelling of polymeric nanoparticles due to hydration, pH and temperature, followed by release through diffusion.
(iii) Dissociation and de-adsorption of the drug from the polymer through a concentration gradient.

Biodegradable polymers most commonly used for the preparation of nanoparticles include homopolymers, such as hydrophilic Polyacrylamide (PAA), hydrophobic poly (lactic acid) (PLA), poly(glycolic acid) (PGA), and poly (caprolactone) (PCL), and copolymers that consist of at least two different monomeric units, such as block and graft copolymers.

##### Homopolymers

PAA, PLA, PGA, and PCL are biocompatible polyester homopolymers commonly used for biomedical applications [112]. Depending on their solubility, homopolymers may be further categorized as water soluble (hydrophilic) or water insoluble (hydrophobic).

PAA is a water soluble polymer formed from acrylamide, C_3_H_5_NO, subunits that is biocompatible to tissues due to its relatively high water content, less toxicity and flexibility of engineering [175]. The neutral surface property of the hydrophilic polyacrylamide-based nano platforms reduces uptake by macrophage and lowers protein adsorption. Its hydrophilic nature further decreases opsonization by plasma proteins in the bloodstream and aids in the evasion of circulating and tissue-based macrophages [176]. PAA also confers greater solubility to hydrophobic PSs to prevent aggregation in the blood stream [177]. Moreover, PAA can be easily copolymerized with other monomers to tune the biodegradation rate or introduce functional groups as targeting tags [178]. Further surface modification enables one to control the behavior of PAA in cells and *in vivo*, more specifically allowing targeting to disease tissues and subcellular organelles [179].

Tang *et al.* synthesized PAA-based nanoparticles for the delivery of methylene blue (MB) PS for PDT and found that the nanoparticles protected the MB from reduction by diaphorase enzymes [175]. *In vitro* studies revealed that the encapsulated MB generated excess singlet oxygen (^1^O_2_), which adequately diffused out of the matrix to damage tumor cells. Moreno *et al.* prepared disulfonated 4,7-diphenyl-1,10-phenantroline ruthenium (Ru(dpp(SO_3_)_2_)_3_)-loaded PAA and amine-functionalized PAA (AFPAA) nanoparticles (monomer: acrylamide and *N*-(3-aminopropyl)methacrylamide) to target cancer cell membranes without entering the cell nor release drugs inside [180]. This strategy avoids back-pumping as well as protects the drugs from being metabolized by lysosomes inside the cells. However, during irradiation of Ru(dpp(SO_3_)_2_)_3_) at 465 nm, singlet oxygen generated in the Probes Encapsulated by Biologically Localized Embedding (PEBBLE) managed to be released from the PAA and AFPAA matrix to react with anthracene-9,10-dipropionic acid (ADPA). Results showed that the polyacrylamide matrix does not quench the ^1^O_2_ produced and allows it to reach the external solution to react with ADPA. In AFPAA, the amount of ^1^O_2_ that reacted with ADPA was slightly reduced to 60% due to slow leaching of Ru(dpp(SO_3_)_2_)_3_. The amine groups on the surface of the particles may be attached with ligands (targeting moieties) for tumor-specific molecular receptors. Systemic delivery of these modified particles would enable tumor-specific localization of these nanoparticles within the tumor mass. It is envisioned that photodynamic activation would be achievable, which would result in the local production of ^1^O_2_ for providing a significant therapeutic benefit.

Lactide is chiral molecule that exists in two optically active forms including the naturally occurring l-lactide isomer and d-lactide [181]. PLA is synthesized either by polycondensation of lactic acid or by ring-opening polymerization (ROP) of lactide, a cyclic diester of lactic acid [182]. The rate degradation of poly (l-lactide) (LPLA) is much slower than that of poly (d,l-lactide) (DLPLA) and requires more than two years to be completely absorbed [183]. Copolymerization of PLLA with d-lactide or by cross-linking PLLA (CL-PLLA) will lead to less stable particles higher rate of degradation [184].

PLA nanoparticles have been used extensively in drug delivery systems for systemic and topical applications to encapsulate hydrophobic compounds and improve solubility limitations [185]. Hypericin (Hy), a natural PS isolated from *Hypericum perforatum*, has been shown, both *in vitro* and *in vivo*, to efficiently detect and treat ovarian cancer [186]. However, due to its hydrophobicity, systemic administration of Hy is difficult. Zeisser-Labouebe *et al.* prepared Hy-loaded PLA nanoparticles and demonstrated the *in vitro* photoactivity of loaded nanoparticles in a NuTu-19 ovarian cancer cell model derived from Fischer 344 rats [187]. Hy loaded in PLA nanoparticles exhibited higher photoactivity compared to the free drug. In addition, at a drug concentration of 0.025 mg/L, Hy loaded in PLA nanoparticles exhibited threefold higher activity compared to the free drug. However, due to its low degradation rate, high crystallinity, and water insolubility, PLA nanoparticles may exhibit slow drug release [188]. To overcome this limitation, researchers have synthesized several co-polymers of lactides including poly (d,l-lactic-*co*-glycolic acid) (PLGA) and poly-(ethylene glycol)-*co*-poly (d,l-lactic) (PEG-PLA) [184,189].

##### Block Copolymer

Block copolymer micelles are formed by the self-assembly of amphiphilic copolymers and exhibit nanoscopic core/shell structure that is useful for the encapsulation of hydrophobic PSs and other hydrophobic drugs through physical, electrostatic, and chemical interactions [190,191,192].

In comparison to PLA, Poly(lactic-*co*-glycolic acid) (PLGA) exhibits faster degradation and potential for controlled and sustained drug delivery [193]. Moreover, the overall physical property of the polymer-drug matrix may be tuned through manipulation of polymer molecular weight, monomer ratio, and drug concentration in order to attain the desirable release kinetics [194]. PLGA is degradable through cleavage of ester bonds by water. However, due to the difference in hydrophobicity of PLA and PGA that arises from the presence of methyl group in PLA, the ratio of PLA to PGA can be tuned to influence the degradation of the copolymer and release of the drug molecule [194]. It is important to note that the relationship between polymer composition and mechanical and degradation properties of the material is not linear. For example, a copolymer of 50% glycolide and 50% d,l-lactide degrades faster than both homopolymers [195]. Size influences nearly every aspect of particle function including degradation, flow properties, clearance and uptake mechanisms [196]. The size of carriers has been shown to govern the passage of drugs from the intra- to the extravascular compartment, namely extravasation [197]. Therefore, PS extravasation can be controlled by the size of NP in which the PS is incorporated [198]. This strategy is also useful for achieving passive targeting of PS to cancer tissues [199]. Vargas *et al.* synthesized m-THPP-loaded PLGA nanoparticles with three different sizes, 117, 285, and 593 nm, using the emulsification-diffusion technique [200]. Nanoparticles with the size of 117 nm exhibited maximum phototoxic effects and highest rate of singlet oxygen production *in vitro* due to enhanced intracellular uptake and greater fraction of particle exposure to surroundings, thereby permitting maximum drug release. Furthermore, a smaller size is correlated to higher photodynamic efficiency and is considered more ideal for PDT cancer treatment due to EPR effect at the cancer tissue. Marchetti *et al.* encapsulated Zinc (II) phthalocyanine (ZnPc) in PLGA nanoparticle using a solvent emulsion evaporation method [201]. After red light treatment at 675 nm, 30% cell viability was noted in neoplastic cells, P388-D1, treated with ZnPc loaded PLGA nanoparticles. In the absence of light, the system exhibited low dark toxicity with cell viability >92%. Other photophysical and photobiological measurements also suggested that ZnPc loaded PLGA nanoparticles are a promising drug delivery system for PDT.

In order to enhance the solubility and polar drug loading capacity of PLA, poly (ethylene glycol) (PEG) has been copolymerized with PLA to form Poly-(ethylene glycol)-*co*-poly(d,l-lactic acid) (PEG-PLA). PEG increases hydrophilicity, enhances drug loading capacity, decreases burst effects, extends the *in vivo* residence time, and evades nanoparticle engulfment by macrophages [202]. In addition PEG has numerous advantages such as flexibility, resistance to immunological recognition and plasma proteins, and biocompatibility [203]. Hydrophobic drugs mainly localize in the hydrophobic matrix of PLA and the drug is either release through diffusion or enzymatic degradation [204]. Drug release from PEG-PLA nanoparticles can be controlled by changing the content of PEG, Mw of PEG, and total Mw of copolymer [205]. Cohen *et al.* synthesized poly-(ethylene glycol)-*co*-poly (d,l-lactic acid) (PEG-PLA) block copolymer by ring-opening polymerization and encapsulated m-THPP into PEG-PLA nanoparticles [206]. PEG-PLA nanoparticles loaded with 5% and 10% of m-THPP resulted in >90% phototoxicity and <20% dark toxicity against HSC-3 and HN-5 cell lines at a micelle concentration of 2–200 µg/mL. Similarly, Bourdon *et al.* studied the cellular uptake, localization, and phototoxicity of meta-tetra (hydroxyphenyl) chlorin (mTHPC) encapsulated in PLA-PEG nanocapsules (NC) and PLA coated with poloxamer 188 (polox PLA NCs) [207]. Cellular uptake by macrophage-like J774, as determined by microspectroflorimetry, is reduced in both NC and polox PLA NCs in comparison to naked PLA, indicating that RES clearance was limited. However, the specific punctate fluorescence in PLA-PEG and difference in diffuse distribution between PLA-PEG and polox PLA NCs suggests different targeting ability. These findings demonstrate that photosensitizers encapsulated in PEG-modified nanocapsules may serve to improve PDT efficacy.

Polycaprolactone (PCL) is a semicrystalline aliphatic polyester synthesized from the relatively inexpensive monomer “Ɛ-caprolactone” through ROP [208]. PCL is mainly used as a drug carrier due to its biocompatibility, biodegradability without the generation of acids such as observed in PLA and PLGA, high permeability to small drug molecules, and ease of blending with other polymers [209]. Due to the slow degradation rate of PCL, several co-polymeric systems, such as (Ɛ-caprolactone)-poly (ethylene glycol) (PECL), have been devised and investigated for improved properties [114]. Ahmed *et al.* prepared vesicles using hydrolysable diblock copolymers of polyethylene glycol-poly-l-lactic acid (PEG-PLA) and polyethylene glycol-polycaprolactone (PEG-PCL) [210]. Doxorubicin, a common anticancer agent, encapsulated within these nanoparticles exhibited comparable loading to that of liposomes. Drug release was accelerated with higher proportion of PEG and delayed when the poly(ester) chain was more hydrophobic (*i.e.*, PCL). The release rate is linearly related to the molar ratio of degradable polymer blended into the membranes consisting of non-degradable polymers. Zhou *et al.* reported HAS-loaded PCL and PECL microspheres prepared by solvent extraction based on the formation of double W/O/W emulsion [211]. Results showed that samples containing 10% and 15% (wt %) PEG exhibited the highest loading efficiency (approximately 50%) among all copolymers and that polymer composition directly affects system properties. Master *et al* prepared PEG-PCL micelles to encapsulate hydrophobic silicon phthalocyanine (Pc4) [212]. *In vitro* studies showed that Pc4-loaded micelles partially localized in the lysosome/endosome while Pc4 solubilized in DMF deposited in the mitochondria, endoplasmic reticulum, and Golgi apparatus. Micelles encapsulating Pc4 exhibited significant cytotoxicity against MCF-7c3 cells after photoirradiation possibly through apoptosis. In another work, Peng *et al.* synthesized amphiphilic 4-armed star-shaped chlorin-conjugated methoxy poly (ethyleneglycol) (mPEG) and poly (Ɛ-caprolactone) (PCL) by reacting the amino groups on chlorin with carboxyl groups on mPEG-b-PCL-COOH [213]. These chlorin-cored star amphiphlic block copolymers self-assembled into micelles and acted both as nano-PS agent for PDT and drug carrier for the hydrophobic drug Paclitaxel. The multifunctional micelles exhibited efficient singlet oxygen generation and improved cytotoxicity against MCF-7 cells due to a synergistic effect contributed by the anti-cancer drug Paclitaxel. Hence, this design demonstrates the potential of synergistic treatment in cancer treatment using photodynamic therapy and chemotherapy.

##### Graft Copolymer

Graft copolymers are polymers or block copolymers with long sequences of one monomer grafted on to the side chain of another [191,214]. Amphiphilic graft copolymers more easily form small micelles due to the lower number of polymer chains that is required when compared to block copolymers. Furthermore, structural variables such as composition, branch length, backbone length, and branch spacing provide vast possibilities for new properties [215]. Xun *et al.* synthesized novel amphiphilic graft copolymers, P(Glu-*alt*-PEG)-*graft*-PCLA, based on poly(ethylene glycol) (PEG) segments and glutamic acid (Glu) units as the hydrophilic main chain, and poly(ɛ-caprolactone-*co*-lactide) (PCLA) as hydrophobic branches [216]. High doxorubicin loading capacity and good controlled release properties resulted in efficiency inhibition of HeLa cells *in vitro*. Moreover, hydrophobic PSs, such as mTHPC, can be encapsulated into the core of the graft copolymer to enhance circulation time and improve tumor selectively [217]. Bourdon *et al.* loaded mTHPC into the core of micelles prepared from poly (d,l-lactic acid) (PLA) grafted with polyethylene glycol (PLA-PEG) [206,209,211,214,216,217]. High drug level was reached shortly after administration to nude mice bearing HT29 human colon tumor while the highest drug level of PLA loaded mTHPC was observed after 72 h. At 24 h post-administration, tumor to muscle ratio of 9 and 10 were estimated for PLA-PEG and polox PLA NCs, respectively, while ratios of 2.6 and 3.2 were found for PLA NCs and standard solution, respectively. The significantly higher tumor accumulation observed during the first hours after administration may be related to long circulation and delayed uptake by the liver. Similarly, Tsai *et al.* synthesized pH-sensitive and non-pH-sensitive graft copolymers, poly (*N*-vinyly caprolactam-*co*-*N*-vinyl imidazole)-g-poly (d,l-lactide) (P (VCL-*co*-VIM)-*g*-PLA) and poly (*N*-vinyly caprolactam)-*g*-poly (d,l-lactide) (P(VCL)-*g*-PLA) for the encapsulation of protoporphyrin IX (PPIX) [218]. *In vitro* result showed that PPIX loaded in pH-sensitive micelles was found in the nucleus, while PPIX loaded in non-pH-sensitive micelles localized within the lysosome. Furthermore, light-specific phototoxicity was observed only when A549 cells were incubated with PPIX-loaded pH-sensitive micelles. This may be due to the difference in localization since ROS can only damage the organelles where PPIX is localized. Cy5.5-modified micelles were injected intravenously through the tail vein of female balb/c nude mice bearing A549 cells xenografts on the back neck to track the distribution of pH sensitive and non-pH sensitive micelles. Results showed that animals treated with PPIX-loaded pH-sensitive micelles exhibited inhibited tumor growth after PDT treatment. These results confirmed that pH-sensitive PDT micelles can efficiently localize to tumors, release PPIX under an acidic environment, produce cytotoxic ROS to induce cell death, and ultimately inhibit tumor growth (1.6- and 2.7-fold inhibition at day 20 for non pH sensitive and pH sensitive micelles, respectively) through vascular damage and immunity effects.

##### Dendrimers and Hyperbranched Polymer

The term dendrimer is derived from the Greek word “*Dendron*” meaning “tree” and is rational in view of their classic structure with numerous branching points [219]. Dendrimers are versatile three-dimensional macromolecules that exhibit compartmentalized structures [219,220]. Dendrimers are highly hydrophilic, biocompatible, polyvalent, and exhibit precise molecular weights [221,222]. A variety of drug molecules can be loaded into dendrimers through electrostatic interactions, simple encapsulation, or covalent conjugation to enhance the solubility of the drug [223,224]. Covalent association may occur through the formation of stable bonds that are cleavable upon reaching the target. Dendrimer disassembly upon temperature or pH triggers can also allow the incorporated drug to be released [225]. Battah *et al.* synthesized a series of novel dendrimers-5-aminolevulinic acid (ALA) using a convergent growth approach where the ALA residues are attached to the periphery of the dendrimers by ester linkages [226]. After treating tumorigenic keratinocyte PAM 212 cell line with ALA ester dendrimers, accumulation was observed in the cells with low dark cytotoxicity, demonstrating potential for delivery of ALA. Kojima *et al.* synthesized and characterized two PEG tethered dendrimers (PEG-PAMAM and PEG-PPI) and encapsulated rose bengal (RB) and protoporphyrin IX (PpIX) PSs [227]. PEG tethered dendrimers were capable of encapsulating more PS and formed stable complexes under the physiological condition. Complex properties also influenced the generation of singlet oxygen, subcellular localization, and the cytotoxic effect, but do not affect photosensitivity acitivty of PpIX. Efficient phototoxicity was observed due to effective production of singlet oxygen and delivery to the mitochondria.

Hyperbranched polymer (HBP) was first coined by Kim and Webster in 1988 and is a highly branched macromolecule with three-dimensional dendritic architecture [228]. HBPs possess properties similar to those of dendrimers, such as weak molecular entanglement, low viscosity, high solubility, and large numbers of functional groups. Due to their unique physical and chemical properties, they exhibit potential applications in various fields ranging from drug delivery to coating [229,230]. Li *et al.* developed, through carbodiimide-mediated reaction between hyperbranched poly (ether-ester) (HPEE) and Ce6, spherical and uniformly dispersed covalent conjugates of HPEE-Ce6 [231]. PDT efficacy was significantly higher when HPEE-Ce6 was used instead of free Ce6 and can be attributed to the higher number of molecules delivered per cell, disaggregating effects contributed by the hydrophilic polymer, and the localization of nanoparticle-bound PS in more sensitive sub-cellular sites.

##### Hydrogel

Hydrogel nanoparticles have gained considerable interest as a suitable drug delivery system due to their ability to contain water, stability in aqueous media, and compatibility with biological systems [232,233]. Among them, hydrogel polyacrylamide (PAA) where the monomeric units are linked together with ester bonds, have been gained great attention for PS delivery due to biocompatibility, biodegradability, and low toxicity [234]. Gao *et al.* synthesized 2–3 nm PAA ultrafine hydrogel nanoparticles and incorporated hydrophobic *meta-*tetra (hydroxyphenyl)-chlorin (*m*-THPC) through a non-aqueous microemulsion method. Ultrafine hydrogel nanoparticles prevented m-THPC aggregation and uptake by the reticuloendothelial system (RES) to enhance its delivery in aqueous media and facilitate singlet oxygen diffusion through the nanoparticle matrix. In an *in vivo* study, PDT using mTHPC-loaded PAA nanoparticles effectively killed rat C6 glioma cells [235]. He *et al.* synthesized a pH, thermal, and redox triple responsive nanogel system (TRN) from poly[(2-(pyridin-2-yldisulfanyl)-*co*-[poly(ethylene glycol)]] (PDA-PEG) and poly (*N*-Isopropyl methacrylamide) (PNiPMA), a thermoresponsive polymer [236]. Pc4-loaded TRNs are stable in physiological condition and swells in acidic pH and high temperature. In the acidic and reducing intralysosomal environment, Pc4 is released. To examine how redox potential affects the transition temperature of TRN, 10 mM DTT was added during the heating process and EDTA was included to eliminate possible intra-particle crosslinking of TRN after DTT treatment. Result showed that the addition of 10 mM DTT/EDTA decreased the transition temperature of TRN from 39 to 36 °C. Since the cytosol has a higher glutathione level compare to the blood, TRN would rapidly self-expand intracellularly. *In vitro* results revealed that 4-Methoxybenzoic acid (MBA) functionalized Pc4-TRN entered UMSC22A cancer cells with the help of sigma-2 receptor, which transfers Pc4 to the mitochondria, and resulted in higher cell toxicity than its non-targeted counterpart. In addition, targetability toward head and neck tumor tissues was confirmed through *in vivo* biodistribution study and MBA-Pc4-TRN was retained at the targeted site for four days.

## 4. Inorganic Nanocarriers

Inorganic nanoparticles may be defined as particles of metal oxide or metallic composition that normally consist of an inorganic core and an organic shell which stabilizes the particle in the biological environment as well as provide functionalization sites for the introduction of biomolecules such as ligands for active targeting [237,238,239]. Inorganic nanoparticles show great potential in drug delivery applications due to their unique physicochemical characteristics [240] such as high surface area per unit volume, fluorescence (e.g., semiconductor “quantum dots”) [241], optical absorption (e.g., metallic nanoparticles [242]), magnetic inimitability (e.g., iron oxide), catalytic properties, easy synthesis, and their ability to be functionalized with moieties and ligands to increase their affinity and selectivity toward receptors and target molecules [243,244,245]. Drugs may be incorporated by physical adsorption or covalent attachment to reactive surface groups and depending on the chemical nature of the therapeutic agent, porous inorganic carriers can provide physical encasement to protect captured drugs from degradation [246]. Furthermore, tuning of size and surface composition has been shown to successfully produce inorganic nanoparticles capable of evading the RES [247].

### 4.1. Drug Loading and Release from Inorganic Nanocarriers

Generally, drug loading methods in/on inorganic nanocarriers are similar to loading in/on organic nanocarrier as discussed above and can be classified into covalent and non-covalent approach [248,249,250,251,252]. Likewise, these common approaches may be further divided into either encapsulation or surface mediated techniques [253]. Drug release from inorganic nanocarrier are also similar to the release from the organic carriers as discussed briefly under organic nanocarriers [254].

### 4.2. Quantum dots

**Figure 5:**
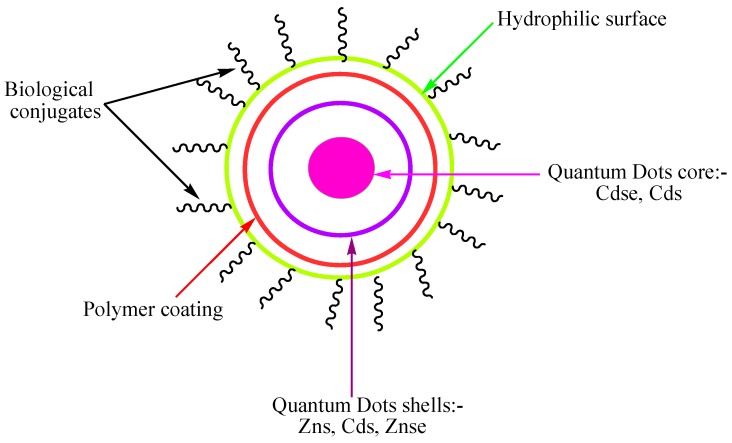
Schematic diagram of quantum dots.

**Figure 6:**
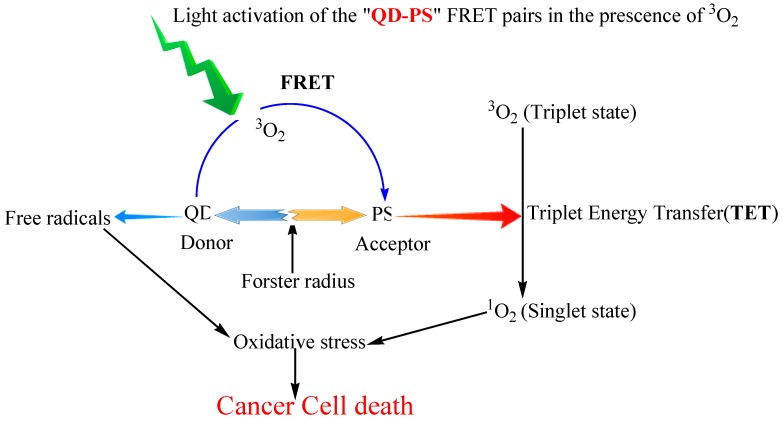
Schematic illustration of PDT using QDs. QD-PS FRET pair confined to the site of cancer is activated by light of a specific wavelength in the presence of molecular oxygen (^3^O_2_) to generate singlet oxygen, which kills cancer cells. Activation of the QD by light can also produce free radicals that play a similar role [268].

Quantum dots (QDs) were first described in 1982 by Efros and Ekimov [255]. QDs are nearly spherical fluorescent semiconductor nanoparticles with a diameter of 2–10 nm that denote a versatile platform for engineering drug delivery vehicles [256,257,258]. The QD structure is illustrated in Figure 5. Due to their unique physical, chemical, and optical properties, in-depth studies may be conducted on nanocarrier interactions with biological systems, biodistribution, intracellular uptake, and drug release [259,260,261]. In addition, their relatively simple and cheap synthesis procedure, cytotoxicity under UV irradiation, high photostability, and tunable fluorescent emission properties make QDs a potential new class of PS carrier [262,263,264]. Further surface modification increases water solubility, biocompatible and target-specificity [265]. In addition, the combination of semiconductor QDs with PSs permits the use of an excitation wavelength that the PS alone does not absorb [265,266]. Samia *et al.* synthesized CdSe QDs linked with silicon Pc photosensitizer (Pc4) through an alkyl amino group on the photosensitizer’s axial substituent [267]. While Pc4 is directly activated between 550 and 630 nm, QD can serve as a primary energy donor to activate Pc4 between 400 and 500 nm. At 488 nm, through a fluorescence resonance energy transfer (FRET) mechanism with an efficiency of 77% from QD to Pc4, the Pc4 emission was observed at 680 nm. (Figure 6) In the process, the team also found that QDs alone generated ^1^O_2_ without a mediating Pc4 molecule. Using CdSe QD with 65% emission quantum yield, they found an ^1^O_2_ quantum yield of ~5% in comparison the ^1^O_2_ efficiency of Pc4 at 43%.

Tsay *et al.* developed a water stable and peptide-coated quantum dot-photosensitizer conjugate using novel covalent conjugation strategy on peptides to overcoat quantum dots (QDs) without affecting the photophysical properties of both the QDs and photosensitizers [269]. Rose bengal and chlorin e6 were covalently attached to phytochelatin related peptides and used to overcoat green- and red-emitting CdSe/CdS/ZnS nanocrystals. This complex was capable of undergoing FRET, which may be particularly useful in extending the range of excitation of PSs to generate singlet oxygen. A 0.31 singlet oxygen quantum yield was recorded using 532-nm excitation wavelength.

### 4.3. Ceramic-Based Nanoparticles

In the last decade, there has been an increasing curiosity in the synthesis of ceramic-based nanoparticles and their potentials in drug delivery [270]. Currently, a variety of synthesis methods are used to reduce the cytotoxicity of ceramic nanoparticles (silica gel) (SiO_2_) [271,272]. Ceramic-based nanoparticles, doped with photosensitive drugs, have a number of advantages over organic polymeric particles such as ease of preparation, controllable properties, and protection of molecules from microbial/enzyme attacks and extreme microenvironment conditions. The preparation process is similar to the well-known sol-gel process and can be used to provide particles with desirable size, shape, and porosity [273,274,275,276,277]. In addition, their surfaces can be easily modified with different functional groups and can be attached to a variety of monoclonal antibodies or other ligands to target them to desired sites *in vivo* [278,279]. Roy *et al.* prepared organically modified silica-based nanoparticles doped with hydrophobic photosensitizing anticancer drug, HPPH [280]. Singlet oxygen was generated after PDT treatment with diode-pumped solid-state laser at 532 nm. Most importantly, silica-based nanoparticles do not affect the absorbance of the entrapped drug and are inert to the therapeutic light used. Similarly, Teng *et al.* synthesized silica-based biocompatible nano-PDT systems that exhibited no dark toxicity and selectivity toward folate receptor presenting cells [281]. These phospholipid-capped mesoporous silica nanoparticles were loaded with PpIX and exhibited remarkable PDT efficacy both *in vitro* and *in vivo*.

Lin *et al.* embedded hydrophobic Tetra-tert-butyl zinc (II) phthalocyanines (Pcs) inside silica nanoparticles and observed an increase in solubility and stability as well ameliorated aggregation [282]. Other studies showed that functionalization of mesoporous silica nanoparticles embedded with hydrophobic PS enhanced PS solubility, decreased aggregation of PSs, and increased the uptake and accumulation of PSs in various cancer cells, ultimately increasing the efficacy of PDT against cancer cells [283,284,285].

### 4.4. Metallic Nanoparticles

Metal nanoparticles are multipurpose agents used in biomedical applications including extremely sensitive diagnostic assays, radiotherapy enhancement and thermal ablation, and gene and drug delivery [286,287]. Metal nanoparticles with maximum absorption and scattering of near infrared radiation (NIR) are also used in PDT of cancer [288]. Gold nanoparticles (AuNPs) are the most studied and commonly metallic nanoparticle used in PDT due to properties such as high surface-to-volume ratio, broad optical properties, chemical inertness, biocompatibility, and the ability to be surface functionalized with various moieties, such as peptides, antibodies, aptamer (ssDNA/RNA oligonucleotide) and biocompatible polymers (e.g., PEG) [289,290,291,292]. In addition, with light treatment at the appropriate wavelength of 700–800 nm, AuNPs will undergo temperature increase, which causes additional damage to tumor cells in a treatment known as hyperthermia therapy. Furthermore, due to localized surface plasmon resonances, AuNPs can be used to increase the excitation efficacy of PSs [293,294]. Wieder *et al.* synthesized hydrophobic phthalocyanines-conjugated AuNPs and found that the normally insoluble phthalocyanine were taken up by HeLa cells and readily generated singlet oxygen when illuminated with light at 690 nm [295]. The combination of nanoparticle and light resulted in 57% and 26% decrease in cell viability when compared to non-light treated phthalocyanine-AuNP and free phthalocyanine, respectively. These properties make phthalocyanine-AuNPs conjugates ideally suited for PDT of cancer.

Cheng *et al.* designed a novel covalent NIR photo-triggerable gold nanoparticles (Au NPs) drug delivery system to effectively deliver photoprecursor Pc 227 in a controlled manner [289]. The Pc227 is covalently bound to AuNPs to produce Pc4 upon photoirradiation at 660 nm. Drug release is based on the photolysis of the axial Si-C bond on the Pc 227 molecule. The release of Pc4 from AuNP behaves differently in aqueous and lipophilic solvents. Greater release was observed in apolar solvents and cell membranes due to the hydrophobic nature of Pc4. In methanol, the photolysis product of Pc227 showed similar singlet oxygen generation ability as Pc4 with 49.0% ± 3.5% quantum yield of the products observed. The system showed no dark toxicity but killed 80% of the cultured HeLa cells upon Vis-NIR.

### 4.5. Carbon Materials

Carbon nanomaterials such as fullerene, carbon nanotube, and graphene oxide, exhibit unique chemical, electrical, and optical properties that draw attention to their application in different areas such as drug delivery, sensors, and biomedical imaging [296,297].

Fullerenes (Buckyballs, soccer-ball-shaped C60) were first observed in 1985 by Robert Curl, Harold Kroto, and Richard Smalley, earning them the Nobel Prize in Chemistry in 1996 [298]. Fullerenes, a new allotrope of carbon, absorbs UV light due to its extended π-conjugated system and produces singlet oxygen species (^1^O_2_), superoxide anions and hydroxyl radicals when excited by light at 532 nm, making fullerenes one of the most important potential candidates for PDT [299,300]. The major limitation of fullerenes, however, is their complete lack of solubility in both the aqueous environment and polar solvents. In order to circumvent these obstacles, fullerenes have been chemically functionalized to confer solubility and versatility [298,301]. Interestingly, fullerenes (C60) alone have the ability to induce cell death, making them potential anticancer and toxic agents [302].Yamakoshi *et al.* showed that oxyl radicals (O_2_*^−^ and *OH) generated from photoexcited fullerenes under physiological conditions are responsible for DNA cleavage, especially in the presence of electron donors like NADH [303]. Similarly, Mroz *et al.* demonstrated that functionalizing C60 with pyrrolidinium groups remarkably enhanced photodynamic activity after light exposure which mediated cancer cell toxicity [300].

Single walled carbon nanotubes (SWNTs) are another category of important carbon nanomaterials that have very high optical absorbance in the near-infrared region. SWNTs functionalized with specific ligands enhanced their binding and internalization to target cancer cells where illumination of nanotubes by NIR radiation activates or triggers cell death without harming normal cells [304]. Kam *et al.* synthesized non-covalently functionalized SWNTs using phospholipids-PEG-folic acid (PL-PEG-FA) [304]. After NIR radiation, extensive cell death in FR^+^ cells was noted while normal cells remained intact. Erbas *et al.* investigated pyrenyl-functionalized distyryl-bodipy sensitizers attached non-covalently to SWNTs and found that singlet oxygen was generated when the system was excited at 660 nm with a red LED array [305].

Another family of carbon based material receiving a significant amount of attention is graphene and their derivatives. Among the graphene derivatives, graphene oxide (GO) has been extensively studied for *in vitro* and *in vivo* drug delivery due to its low cost, scalable production, simple functionalization, high solubility, and stability in biological solutions. [306,307]. Huang *et al.* designed a novel folic acid-conjugated graphene oxide (GO) and loaded PS Chlorin e6 (Ce6) via hydrophobic interactions and π-π stacking [308]. They found that the uptake of FA-GO-Ce6 significantly increased through interaction with folate receptors and Ce6 PS was released and accumulated in the cytoplasm due to change in pH as endosomes were turned into lysosomes. *In vitro* study using MGC803 cells shows a remarkable photodynamic efficacy of FA-GO-Ce6 that was dependent on the ratio of mFA-GO/mCe6. When the ratio of mFA-GO/mCe6 was above 2:1 there was no dark toxicity and greater than 80% cell viability was observed. When the ratio of mFA-GO/mCe6 reached 1:1, cell viability was less than 50%. These results indicated that toxicity is dependent on the concentration of Ce6. These folic acid-conjugated GO loaded Ce6 displays great potential as an effective drug delivery system tunable through drug concentration.

## 5. Side Effects of PDT

There are no pronounced side effects of PDT but some arise due to sensitivity to light and undesirable biodistribution of PSs to normal cells [309,310]. Most photosensitizing agents do not remain concentrated in the skin but low concentrations may be retained for several weeks, such as after Photofrin use [311]. Side effects may include burning/stinging sensation, swelling, redness, crusting, itchiness, peeling, blisters, and skin infections [312,313,314].

## 6. Conclusions

Photodynamic therapy is unquestionably a highly effective therapeutic alternative for the treatment of cancers. However, due to limitations of PSs, their full potential has not been realized and their approval as a first line treatment of many cancers has been delayed. Since the majority of first and second generation PSs have drawbacks such as poor solubility in the physiological environments, adverse pharmacokinetics, and poor tumor selectivity, nanoparticle formulations provide a solution to overcome the mentioned limitations. Biocompatible nanoparticles, both organic and inorganic, have been developed as novel PSs carriers. In addition, surface functionalization and modification with targeting ligands could further augment the selective accumulation of PSs loaded nanoparticles at the target site.
